# A Multispectral Photon-Counting Double Random Phase Encoding Scheme for Image Authentication

**DOI:** 10.3390/s140508877

**Published:** 2014-05-20

**Authors:** Faliu Yi, Inkyu Moon, Yeon H. Lee

**Affiliations:** 1 Department of Computer Engineering, Chosun University, 309 Pilmun-daero, Dong-gu, Gwangju 501-759, Korea; E-Mail: yifaliu@chosun.kr; 2 School of Information and Communication Engineering, Sungkyunkwan University, Suwon, Kyongkido 440-746, Korea; E-Mail: yeonlee@ece.skku.ac.kr

**Keywords:** optical security and encryption, pattern recognition, multispectral photon counting imaging, double random phase encryption, nonlinear correlators, color images

## Abstract

In this paper, we propose a new method for color image-based authentication that combines multispectral photon-counting imaging (MPCI) and double random phase encoding (DRPE) schemes. The sparsely distributed information from MPCI and the stationary white noise signal from DRPE make intruder attacks difficult. In this authentication method, the original multispectral RGB color image is down-sampled into a Bayer image. The three types of color samples (red, green and blue color) in the Bayer image are encrypted with DRPE and the amplitude part of the resulting image is photon counted. The corresponding phase information that has nonzero amplitude after photon counting is then kept for decryption. Experimental results show that the retrieved images from the proposed method do not visually resemble their original counterparts. Nevertheless, the original color image can be efficiently verified with statistical nonlinear correlations. Our experimental results also show that different interpolation algorithms applied to Bayer images result in different verification effects for multispectral RGB color images.

## 1. Introduction

Double random phase encoding (DRPE) and its applications have been extensively studied for image encryption, information hiding and watermarking [1–5]. DRPE has the advantage of converting input data into stationary white noise images that do not reveal any information related to the primary image. The original image can be extracted only when appropriate keys are given. Even though the DRPE algorithm has performed well in the image security field [6,7], it has been reported that DRPE is vulnerable to chosen-cipher text attacks [8,9]. Therefore, the encryption keys used in DRPE could be reproduced by an intruder who has repeatedly accessed the DRPE system. In order to enhance the security of DRPE systems, many improvements have been proposed [10–15]. One of the methods presented by Pérez-Cabré *et al.* [10,11] has proven its robustness against unauthorized attacks by integrating a photon-counting imaging (PCI) technique [10,11] with the conventional DRPE approach. PCI can produce image data with sparse distribution. The output image from the system that combines DRPE with PCI, reported in [10,11], does not resemble its input image and cannot be visually distinguished from its counterpart which can safeguard DRPE from unauthorized attacks and improve its security to some extent. Even so, the decrypted image can be verified with a nonlinear correlation method [10,11,16,17]. This method is not intended for visualizing the input image, but for authenticating the original image. However, the promising method reported in [10,11] was tested only for binary and monochrome images. Color images, which can provide a variety of object information to people, now exist universally and are used widely for object visualization, recognition and encryption [18–23]. Consequently, developing an authentication method based on multispectral color images is essential and significant. Recently, we have demonstrated that it is possible to implement a multispectral photon counting integral imaging system using Bayer elemental images for multispectral visualization of photon-limited 3D scenes [24]. As an extended work, it may be possible to apply this multispectral photon counting imaging (MPCI) technique to the verification of multispectral color images by combining it with a DRPE algorithm based on Bayer images, which can be converted into RGB color images with demosaicing methods [25–27].

In this study, we show that the integration of MPCI and DRPE can be used for the authentication of multispectral images. In this procedure, the three color samples (pixels of red, green, and blue color [24]) in Bayer images which have been down-sampled from a primary RGB color image are encrypted with DRPE and the amplitude part of the resulting image is photon counted. The corresponding phase information having nonzero amplitude after photon counting is then retained for decryption. For authentication, the retrieved image from the integration is verified with a statistical nonlinear correlation approach [10,11]. In order to show that the interpolation algorithm used to transform a decrypted Bayer image into a RGB image affects the verification of multispectral RGB images, various interpolation approaches are compared based on nonlinear correlation results. Our proposed approach has introduced multispectral photon counting technique into traditional encryption algorithm. Compared with other color encryption methods [18–23], the scheme combining MPCI and DRPE in this paper can make the decrypted image visually unrecognized under a low light level while the decrypted image can be authenticated properly with nonlinear cross correlation.

This paper is organized as follows: in Section 2, we describe double random phase encryption. In Section 3, the concept of multispectral photon-counting imaging techniques is explained. In Section 4, we present the procedure for the combination of MPCI and DRPE. Section 5 includes the experimental results. We conclude the paper with Section 6.

## 2. Double Random Phase Encoding (DRPE)

**Figure 1.**
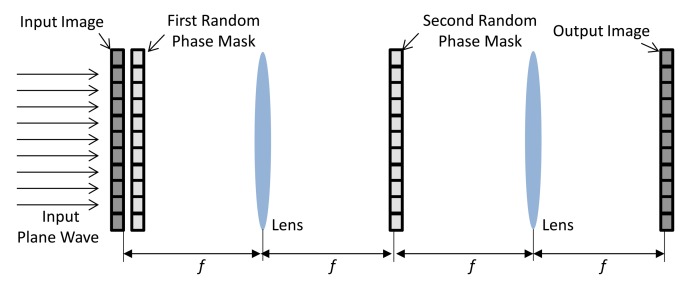
Schematic diagram of the DRPE system (*f* is the focal length of the lens).

Optical and digital information security systems [28–35] based on the double random phase encoding (DRPE) [25–30,30–32] technique has played a predominant role in information security. According to the DRPE principle, the primary image *f*(*x*, *y*), which represents the spatial coordinates of a two-dimensional signal or image, is encrypted into stationary white noise data using two random phase masks. The two random phase masks in the spatial and frequency domains are expressed as exp(*j*2*πn*(*x*, *y*)) and exp(*j*2*πb*(*μ*, *ν*)), with *n*(*x*, *y*) and *b*(*μ*, *ν*)uniformly distributed over (0,1). These two domains are statistically independent. The encryption process of the double random phase algorithm can be described as follows [1]:

(1) $$f_{c}(x,y)={\text{J}}^{-1}\left[\text{J}\left[f(x,y)\exp(j2\pi n(x,y))\right]\exp[j2\pi b(\mu ,\nu )]\right]$$

where 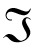 and 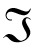
^−1^ denote a two-dimensional Fourier transform and an inverse Fourier transform, respectively. For decryption, the procedure is reversed. The DRPE schematic in the Fourier domain is given in Figure 1.

## 3. Multispectral Photon Counting Imaging (MPCI)

**Figure 2.**
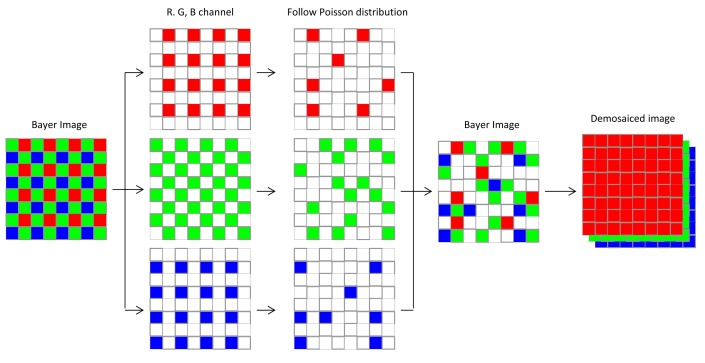
The schematic diagram of MPCI.

Photon-counting imaging, a special class of optical imaging techniques, has been successfully applied in fields such as 3D imaging and 2D/3D object recognition in photon-limited situations [36–39]. Photon-counting imaging systems are designed for low light levels (photon-starved conditions) or night vision, situations in which only a limited number of photons reach the image sensors [36–39]. Monochromatic photon counting imaging could be achieved by allowing only a limited number of incident photons to the captured image scene. This scheme includes the assumption that the probability of counting photons at any arbitrary pixel in a captured image follows a Poisson distribution [10,36–39]. For MPCI in low light levels, a similar approach might be used to control the expected number of photons in the Bayer image, which is captured by a color image sensor with a color-filter array (CFA), known as a Bayer CFA. In the Bayer image pattern, the green samples (luminance-sensitive elements) are arranged in a checkerboard pattern, and red and blue samples (chrominance-sensitive elements) are arranged in a rectangular pattern. In the MPCI scheme, it is assumed that the Bayer image is decomposed into red, green, and blue channels. The probability of counting *l_w_*(*x*, *y*) photons at any arbitrary pixel point (*x*, *y*) on each spectral channel can be modeled with a Poisson distribution as follows [24]:

(2) $$\text{Poisson}(\lambda_{w}(x,y)={\bar{f}}_{B,w}(x,y)\times N_{p})=\frac{{\left[\lambda_{w}(x,y)\right]}^{l_{w}(x,y)}e^{-\lambda_{w}(x,y)}}{l_{w}(x,y)!}$$

where the subscript *w* denotes red, green, or blue colors, *λ_w_*(*x, y*) is the Poisson parameter at any arbitrary pixel point (*x*, *y*) on each spectral channel that is computed by *λ_w_*(*x, y*) = *f̄_B,w_*(*x, y*) × *N_p_* while *N_p_* is the expected number of photons in the Bayer image and *f̄_B,w_*(*x, y*) is the normalized irradiance at pixel points (*x, y*) on each spectral channel as follows [24]:

(3) $${\bar{f}}_{B,w}(x,y)=\frac{f_{B,w}(x,y)}{\overset{M}{\underset{x=1}{\text{∑}}}\overset{N}{\underset{y=1}{\text{∑}}}f_{B}(x,y)}$$

where *f_B_*(*x*, *y*) is a Bayer patterned image, and *M* and *N* are the total numbers of pixels in the Bayer image in the *x* and *y* directions, respectively. Then, the photon-limited Bayer image for each color channel *f_ph_*(*x*, *y*, *w*) is obtained as follows:

(4) $$f_{ph}(x,y,w)=\text{Poissrnd}(\lambda_{w}(x,y)=N_{p}\times {\bar{f}}_{B,w}(x,y))$$

where *Poissrnd*(•) is a function to generate random numbers from the Poisson distribution with Poisson parameter *λ_w_*(*x*, *y*). Finally, MPCI can be achieved by estimating the missing two color samples in the generated photon-limited image that has a Bayer format using demosaicing algorithms [25–27]. In this paper, the gradient-corrected linear interpolation technique recently proposed by Malvar [25], which is an adaptive interpolation algorithm, is employed to attain MPCI at low light levels through demosaicing of Bayer-patterned, photon-limited images. The schematic diagram of MPCI is described as Figure 2.

## 4. Integration of MPCI and DRPE

Though DRPE on its own is vulnerable to chosen-plaintext and chosen-cyphertext attacks [8,9], integrating DRPE with PCI has exhibited robust resistance against intruder attacks based on binary and monochrome images [10,11]. In this paper, we extend the research in [24] and combine DRPE with the MPCI technique for multispectral color image authentication. The detailed procedure for the combination is given below.

**Figure 3.**
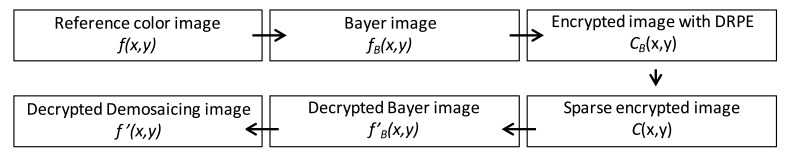
Flow diagram of the proposed color image authentication method.

**Figure 4.**
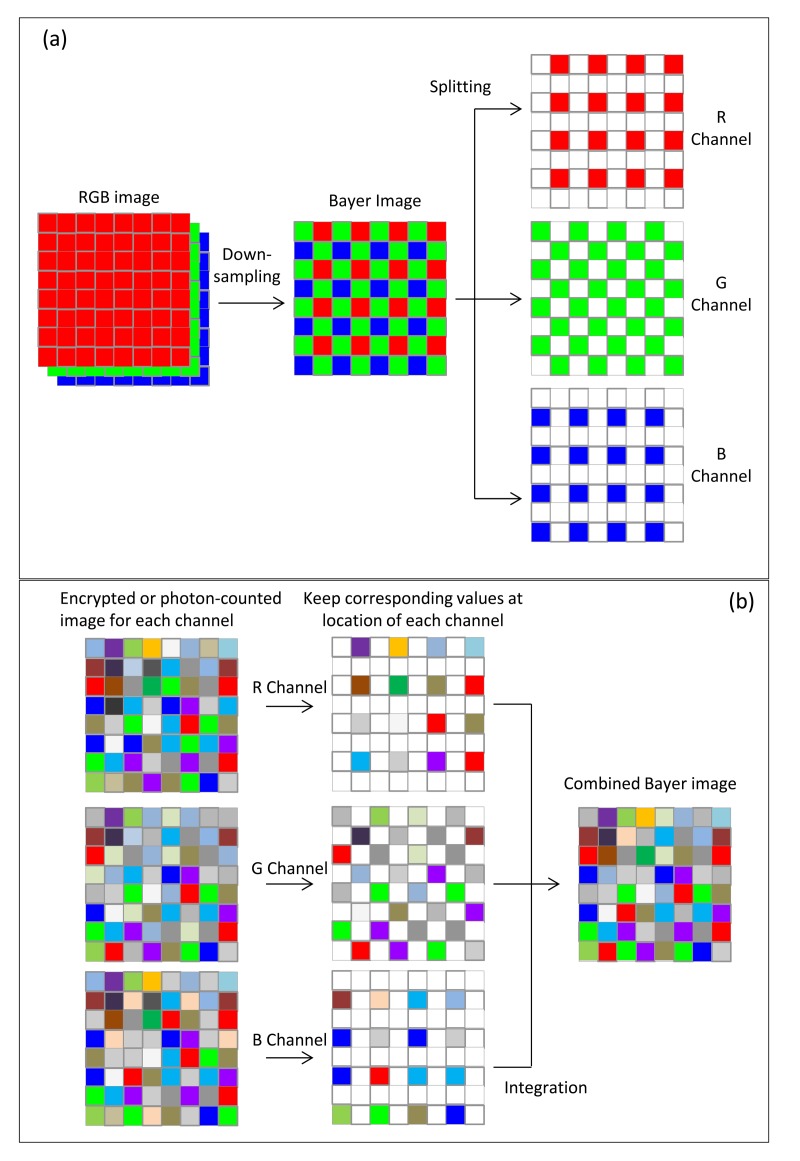
Illustration of down-sampling, image splitting and integration. (**a**) Illustration of down-sampling (from RGB image to Bayer image) and image splitting (from Bayer image to R, G, B channel); (**b**) Illustration of image integration (from R, G, B channel to Bayer image). The pixel in white denotes zero value.

The input multispectral color image *f*(*x,y*) is first down-sampled to form a Bayer-pattern image *f_B_*(*x,y*) [25]. Then, the three types of color samples (red, green and blue channel) in the Bayer image are encrypted individually with DRPE sharing the same keys with the same size and resulting in an image with the distribution of stationary white noise. The three encrypted images are then combined into a new Bayer image denoted by *C_B_*(*x,y*), and each pixel value would be a complex number including amplitude and phase information. Since the phase information should be used in the decryption process, they cannot all be discarded. If they were, it would be impossible to recover the information of the original image. In this procedure, the multispectral photon-counting technique is applied to the amplitude image *A*(*x,y*) of the encrypted Bayer image *C_B_*(*x,y*) obtained from DRPE. As described in Section 3, the three color samples of amplitude image *A*(*x,y*) in *C_B_*(*x,y*) are photon-counted individually. PCI makes some of the pixel values in *A*(*x,y*) zero, and we only keep the phase information for those pixels with non-zero amplitude value. The photon-counted images from the three channels are integrated as one encrypted image *C*(*x,y*). This encrypted image will be sparsely distributed and it can be suitable for compression, which reduces the bandwidth needed for data transmission. For decryption, the red, green, and blue samples in the sparse encrypted image *C*(*x,y*) undergo double random phase decryption individually, forming a combined, decrypted Bayer image *f′_B_*(*x,y*) that looks like a noisy image and, obviously, cannot be visually recognized by the human eye. Finally, the decrypted demosaiced image *f′*(*x,y*) derived from the decrypted Bayer image *f′_B_*(*x,y*) with an interpolation method can be compared with the reference color image *f*(*x,y*) using nonlinear correlation analysis to achieve the authentication function. A schematic representation of this procedure is shown in Figure 3. The detail operations for down-sampling, image splitting and channel integration mentioned in this section are further illustrated in Figure 4. All of the Bayer and channel images are two-dimensional with the same size as that of original RGB colorful image in *x* and *y* axis. The “GRBG” alignment is used for the Bayer pattern. For down-sampling, the pixel values in Bayer image are extracted from the values of corresponding channel in RGB image while other two channel values are discarded. For example, the value of first pixel in Bayer image is the value of the corresponding pixel in RGB image at G channel and the values at R and B channels are ignored (see Figure 4a). Since each channel in Bayer image is encrypted and photon-counted individually, it means the image splitting is necessary. The image splitting process can be demonstrated in Figure 4a. Each channel image is extracted from Bayer image and the values at location of corresponding channel in Bayer image are kept while values at other locations are set to be zero (see pixel in white in Figure 4a). When each channel is encrypted with DRPE or photon-counted, we only utilize the values at the location with the same component and the other values are given to be zeros. Consequently, the three channel images can be added into one Bayer image as showed in Figure 4b.

## 5. Numerical Results

In this paper, all of the results are obtained from numerical simulation using virtual optics on Matlab (R2010a) that is executed on a 32-bits window 7 OS computer which includes an Intel Core i5-2500k processor of 3.3 GHz and the RAM is 4 GB. The quantization level is 256 for the original and output image. Also, all of the processing data are digitally recorded on computer without optical configurations. Since the decrypted images from the proposed procedure are not visually recognizable with a limited number of photons, it is necessary to adopt some comparison scheme to authenticate the retrieved image. In this paper, nonlinear cross-correlation [10,11,16,17] is used to compare the decrypted image with the reference multispectral image. The nonlinear cross-correlation *cc*(*x*, *y*) between the reference image and the decrypted image produced from the input test image is defined as follows:

(5) $$cc\left(x,y\right)={\text{J}}^{-1}\left\{{\left|D(\mu ,\eta )F(\mu ,\eta )\right|}^{k}\exp\left[i\left(\phi_{D}(\mu ,\eta )-\phi_{F}\left(\mu ,\eta \right)\right)\right]\right\}$$

where *D*(*μ*, *η*) and *F*(*μ*, *η*) are 2D Fourier transforms of the decrypted and reference images, *ϕ_D_*(*μ*, *η*) and *ϕ_F_*(*μ*, *η*) are the phase parts of *D*(*μ*, *η*) and *F*(*μ*, *η*), respectively, and parameter *k* defines the strength of the applied nonlinearity. When *k* = 0, the nonlinear cross-correlation equation is the phase extractor leading to enhance the high frequency content and *k* = 1 would let the equation degenerate to a linear filtering method [10,11]. A different parameter *k* would result in different cross-correlation values. The appropriate parameter *k* can be found by analyzing the best peak-to-correlation energy (PCE) result, following equation [10,11]:

(6) $$PCE=\frac{\max\left[|cc(x,y){|}^{2}\right]}{\overset{M}{\underset{i=1}{\text{∑}}}\overset{N}{\underset{j=1}{\text{∑}}}\left|cc{\left(x_{i},y_{j}\right)}^{2}\right|}$$

where *cc*(*x_i_,y_j_*) is the nonlinear cross-correlation result between the decrypted image and the reference image, and *M* and *N* are the image sizes along the *x* and *y* axes, respectively. Since PCE is defined as the ratio between the maximum intensity peak value and the total energy of the nonlinear cross-correlation image, a higher PCE value implies a good nonlinear cross-correlation result.

**Figure 5.**
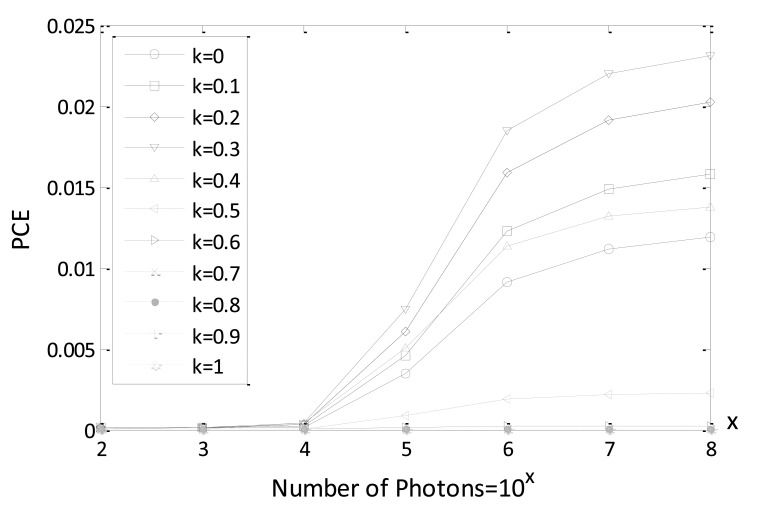
PCE values with various *k* values using true class image (Lena Bayer image).

In Figure 5, PCE values are given, varying the expected number of photons for different parameters *k* using reference and decrypted Bayer images (true class). We note, from Figure 5, that PCE values increase with an increase in the number of photons. Especially, when parameter *k* is within a range from 0.2 to 0.4 better PCE values are achieved for true-class images. Consequently, *k* = 0.3 is selected as an intermediate value for all of the following simulations. It is also noted that 10^4^ is the watershed for the number of photons to achieve a better nonlinear correlation plane with sharp peak. It is verified in the following simulation that the information authentication for true class image can also be realized for Bayer image when the number of photons is equal to 10^4^.

**Figure 6.**
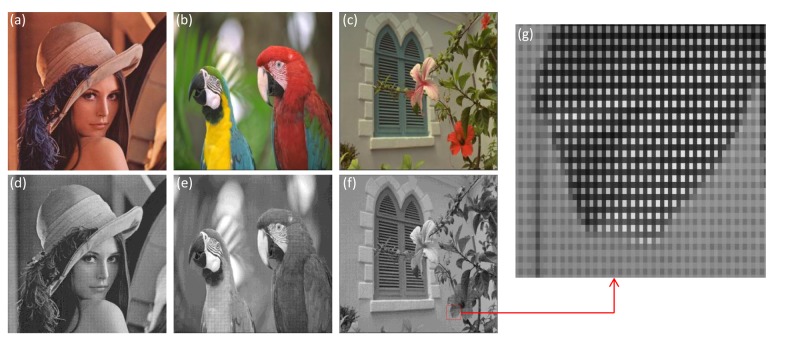
Reference multispectral images and the correspoding down-sampled Bayer images. (**a**) Lena image; (**b**) Parrots image; (**c**) Flowers image; (**d**) Down-sampled Bayer image of Lena; (**e**) Down-sampled Bayer image of Parrots; (**f**) Down-sampled Bayer image of Flowers; (**g**) The enlarged Bayer image for portion of image (f).

In this simulation, three standard multispectral RGB color images taken from Kodak true color image data sets were used and are shown in Figure 6a–c. Each color image has dimensions of 512 × 512 × 3. The proposed integration for color image authentication is conducted with Bayer images and multispectral RGB images. The three corresponding Bayer images including three channels are given in Figure 6d–f.

**Figure 7.**
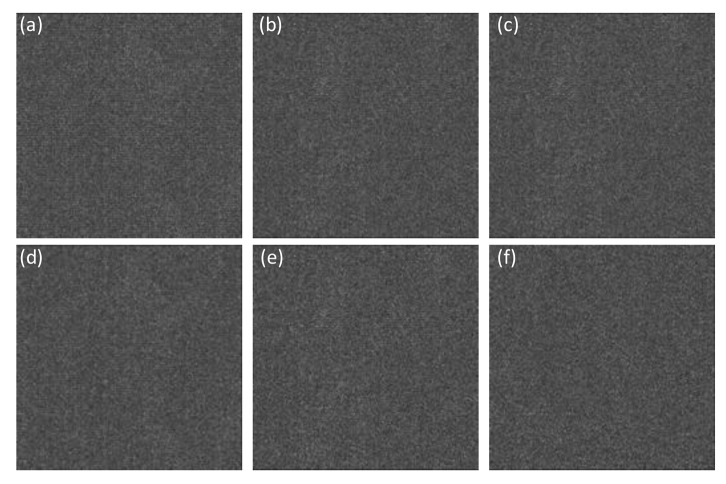
(**a**) Decryped Bayer image of Lena; (**b**) Decryped Bayer image of Parrots; (**c**) Decryped Bayer image of Flowers; (**d**) Decrypted multispectral image of Lena; (**e**) Decrypted multispectral image of Parrots; (**f**) Decrypted multispectral image of Flowers. (k = 0.3 and number of photons = 10^4^).

The down-sampled Bayer images were encrypted with DRPE and then the three color samples of amplitude image in the Bayer image encrypted by DRPE were photon-counted individually to obtain the sparse encrypted image. In order to evaluate the proposed authentication method's performance, the sparse encrypted image was decrypted in Bayer format and corresponding decrypted multispectral color image was obtained from Malvar's demosaicing approach [25] as shown in Figure 7. Here, the photon numbers used in MPCI is 10^4^ (3.8% of total image pixels). Note from Figure 7 that the decrypted color images from the proposed procedure are noisy. Obviously, the retrieval images from the procedure can not be visually recognized with a limited number of photons. However, this procedure can still be used for multispectral image authentication.

**Figure 8.**
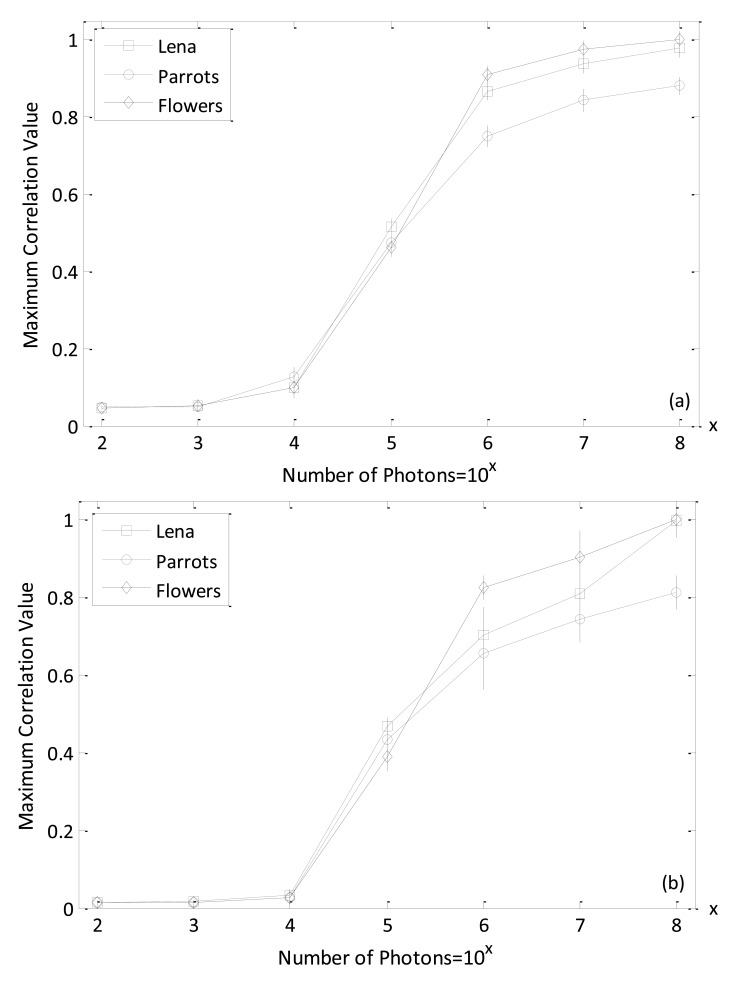
(**a**) The maximum correlation values between reference and decrypted true-class Bayer images; (**b**) The maximum correlation values between reference and decrypted demoasicing RGB true class images. (Error bars represent ±1 standard deviation of the 50 times measurement, k = 0.3).

Figure 8 shows the maximum-intensity cross-correlation values between reference and decrypted true class Bayer and the reference and demosaicing multispectral images with varied number of photons. Here, the parameter value *k* in Equation (5) is set at 0.3, which is verified in the data presented in Figure 5 as being suitable for obtaining reasonable discrimination results. For calculating the intensity cross-correlation values with the Bayer image, the decrypted true class Bayer images are cross-correlated with the reference image (remember, the Bayer images were down-sampled from the reference color image). For the computation of intensity cross-correlation values with the multispectral RGB color image, each channel between the demosaiced true-class color image obtained from the decrypted Bayer image and the reference multispectral RGB color image is cross-correlated. Then the average values of the three cross-correlation values resulting from the three channels (red, green, and blue) are measured to determine the correlation values of multispectral image pairs. The maximum nonlinear cross-correlation values in Figure 8 demonstrate that the photon-limited encrypted image can achieve a good authentication performance. Furthermore, the results indicate that the maximum correlation values tend to increase when the number of photons is more than 10^3^ and 10^4^ for true-class Bayer and demoasiced image, respectively. In this paper, all the maximum nonlinear cross-correlation values are an average of 50 numerical simulations and are normalized as a whole.

**Figure 9.**
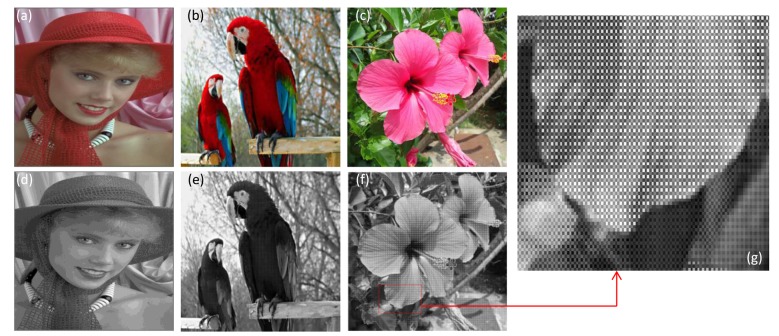
Non-authorized (false class) multispectral images and the correspoding down-sampled Bayer images (**a**) Non-authorized image (false class I); (**b**) Non-authorized image (false class II); (**c**) Non-authorized image (false class III); (**d**) Down-sampled Bayer image of the false class I; (**e**) Down-sampled Bayer image of the false class II; (**f**) Down-sampled Bayer image of the false class III; (**g**) The enlarged Bayer image for portion of image (f). (k=0.3).

The simulation results, presented in Figure 8, have shown that reasonable nonlinear cross-correlation values can be obtained for authorized (true class) multispectral images. To validate the discrimination capability of the proposed procedure, the nonlinear cross-corrrelation results with non-authorized (false class) multispectral images was also tested. The images presented in Figure 9a–c are false-class color images and were used to verify our system. The corresponding down-sampled Bayer images including three channels are given in Figure 9d–f.

**Figure 10.**
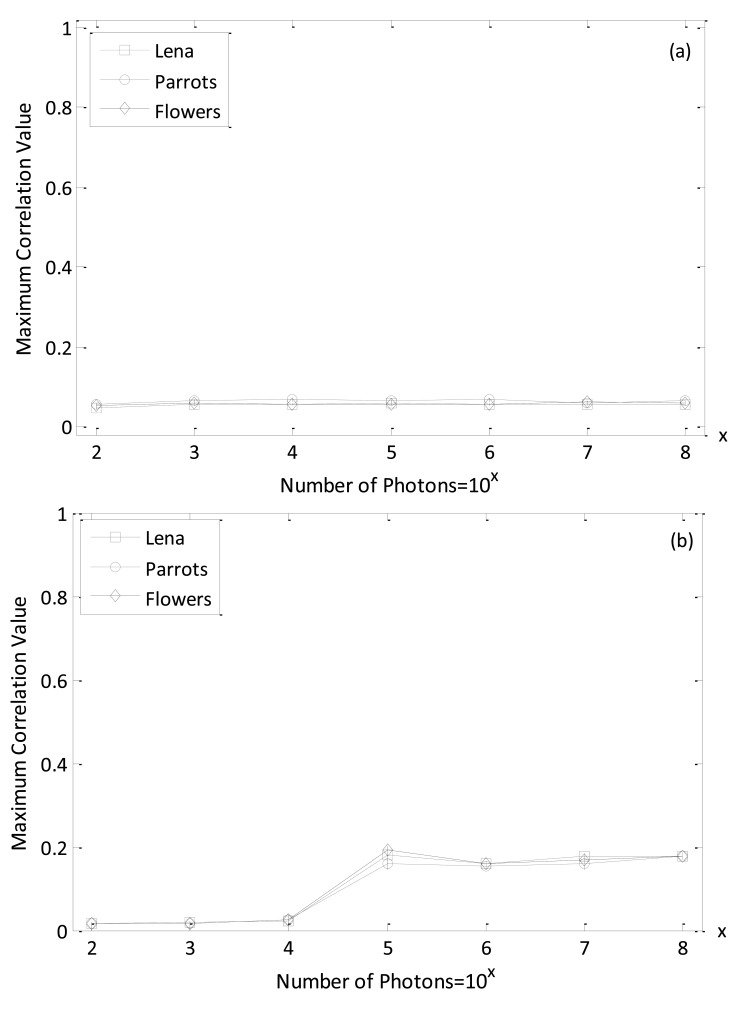
(**a**) The maximum nonlinear cross-correlation values between reference Bayer and decrypted false class Bayer images; (**b**) The maximum nonlinear cross-correlation values between reference and decrypted false class multispectral images. (Error bars represent ±1 standard deviation of the 50 times measurement, k = 0.3).

**Figure 11.**
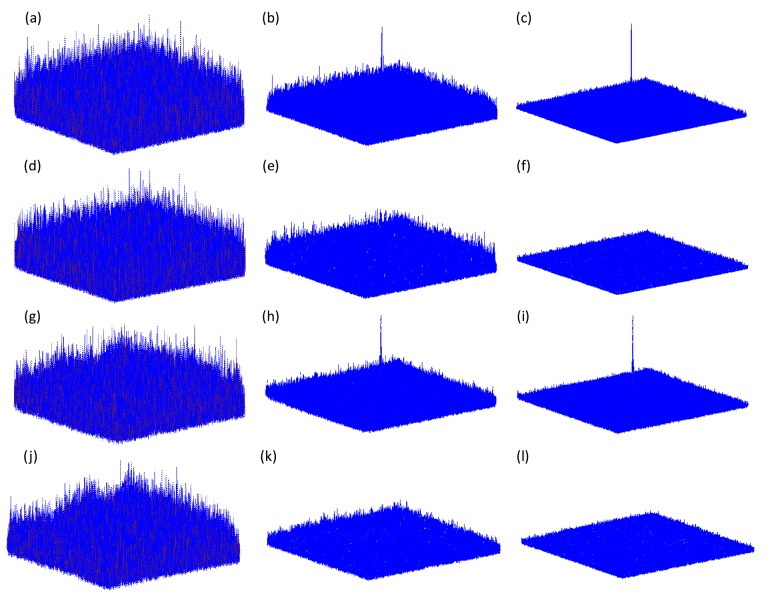
(**a**–**c**) Nonlinear correlation planes between reference and decrypted true class Bayer images with number of photons 10^3^, 10^4^ and 10^5^ respectively; (**d**–**f**) Nonlinear correlation planes between reference and decrypted false class Bayer images with number of photons 10^3^, 10^4^ and 10^5^ respectively; (**g**–**i**) Nonlinear correlation planes between reference and decrypted true class multispectral images with number of photons 10^4^, 10^5^ and 10^6^ respectively; (**j**–**l**) Nonlinear correlation planes between reference and decrypted false class multispectral images with number of photons 10^4^, 10^5^ and 10^6^ respectively. (k = 0.3).

Figure 10 shows the maximum nonlinear cross-correlation values between reference images in Figure 6 and the corresponding false-class images given in Figure 9, with both Bayer and multispectral images. It can be seen that all of the maximum nonlinear correlation values are very small, even when the number of photons is increased. Comparing the true-class image maximum nonlinear cross-correlation values shown in Figure 8 with those derived from false-class images and shown in Figure 10, we note that our method can appropriately distinguish authorized (true class) and non-authorized (false class) multispectral images. This is because the maximum correlation values for true class images are larger than those for false class images. In addition, it is noted from Figure 8 and Figure 10 that when the number of photons is more than 10^4^, the image verification can be achieved between referennce and decrypted Bayer images because the maximum correlation values between the true and false images start to be especially distinct. However, the number of photons have to be at least 10^5^ so as to succesfully realize information authentication for multispectral demosaiced images. This may be explained that the demasicing technique can not exactly recover the missing color component in Bayer image. In Figure 11, the nonlinear correlation planes are given to better visually show the effect of image authentication and verify the previous analysis that 10^4^ and 10^5^ are the watershed number of photons for Bayer and multispectral demosaiced images in terms of correct authentication. Here, we recommend that it is better to make the photon number more than 10^4^ for Bayer image authentication in the real situation since the energy of correlation peak in Figure 11b is not much bigger than that in Figure 11e.

**Figure 12.**
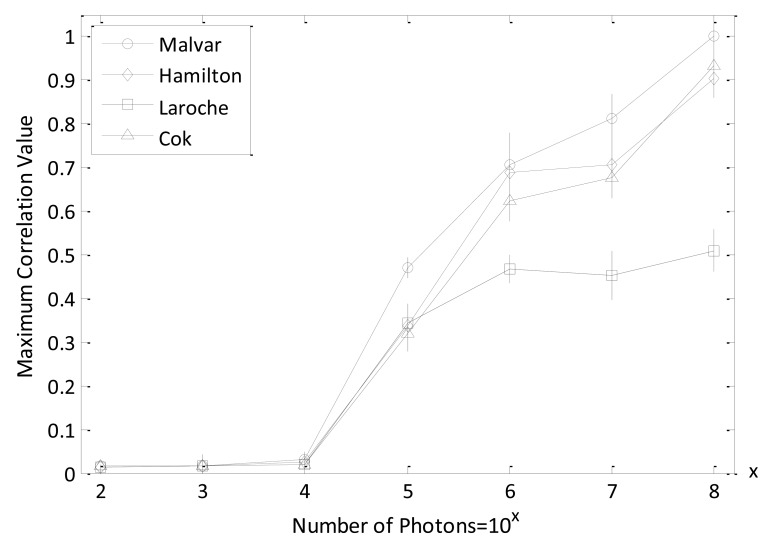
Maximum nonlinear cross-correlation values with different demosaicing techniques using Lena color image. [Error bars represent ±1 standard deviation of the 50 times measurement, (k = 0.3).

Since the multispectral RGB color image obtained from the decrypted Bayer image is related to demosaicing (interpolation) algorithms, different demosaicing approaches will change RGB quality and interact with the nonlinear cross-correlation values. In Figure 12, the maximum nonlinear cross-correlation values for frequently used interpolation methods (Malvar's, Hamilton-Adams's, Laroche-Prescott's, and Cok's) [25–27] with varying number of photons are plotted. It has been demonstrated that Malvar's interpolation method can attain the best nonliear cross-correlation value compared to other demosaicing algorithms, which is consistent with the results shown in [24]. When the numbers of photons are more than 10^5^, the advantage is especially striking. In this study, we adopted Malvar's method as the demosaicing scheme to convert Bayer images into multispectral RGB color images. Malvar's demosaicing algorithm is a gradient-corrected linear interploation technique that can convert a Bayer-format image into a true-color (RGB) image for each pixel format by estimating the missing pixel values on the Bayer image with the help of interpolated current pixel values and the calculated gradient information. The combination of gradient values and linear interploation in Malvar's method can improve the image quality as compared to other interpolation algorithms [24].

**Figure 13.**
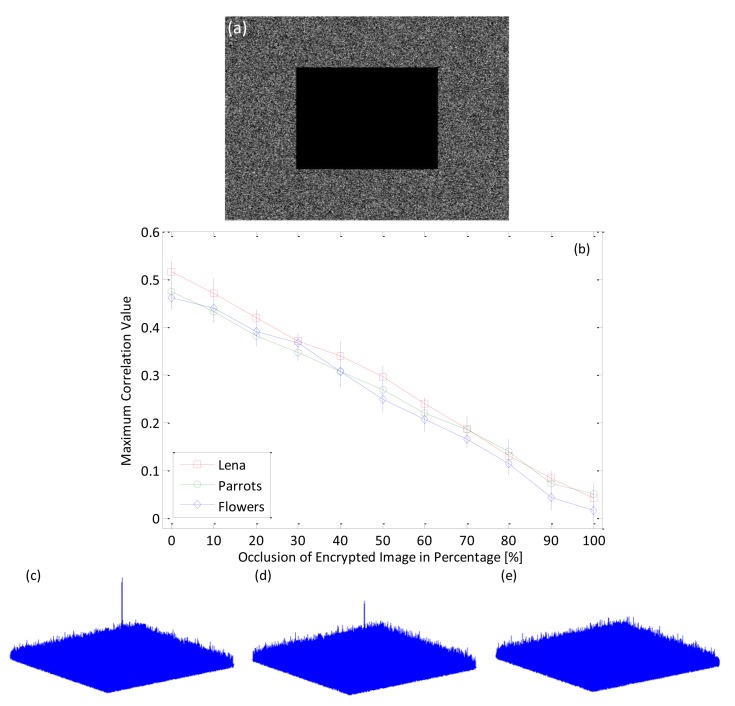
(**a**) Illustration of about 30% occlusion in encrypted image (values at occlusion area is set to be zero); (**b**) Maximum nonlinear correlation values with varying occlusion percentage in the encrypted image (Error bars represent ±1 standard deviation of the 50 time measurements, k = 0.3); (**c**–**e**) nonlinear correlation planes for true class Lena Bayer image when the encrypted image is occluded around 70%, 80% and 90% of the image area, respectively (k = 0.3, number of photons = 10^5^).

The proposed procedure that integrate MPCI and DRPE can be robust even when the encrypted image has been occluded. When some parts of the encrypted image obtained from the procedure have been changed or removed, the image can still be authenticated with a nonlinear cross-correlation technique. Figure 13 shows the maximum nonlinear cross-correlation values with number of photons 10^5^ between reference color (Lena color image) image and the decrypted true-class color (Lena color image) image that is derived from the encrypted image, but where some portion of the pixels have been occluded and changed.Even when about 80% of the original pixel values in the encrypted images are occluded, the input images can still be authenticated, since the corresponding correlation values are larger than those from false-class images, as shown in Figure 10. This can also be visually demonstrated by using the nonlinear correlation planes as shown in Figure 13c–e. It is noted that when the occluded area is less or equal to 80% of the original pixel number, the correlation plane with sharp correlation peak is achieved which verify that our proposed algorithm is effective to the occlusion in encrypted image,even the occlusion reach to 80% of the image area.

It is clear that an ideal image encryption scheme should be sensitive to its key, meaning that when a small number of key values are changed, the decrypted image should be totally different from an image decrypted using the correct key. The nonlinear cross-correlation values between the reference Bayer image and the decrypted Bayer image with the key values partially changed are calculated to be around 0.039 with the number of photons equal 10^5^ and parameter k = 0.3, even when the proportion of changed key values is varied from 1% to 100%. In this numerical simulation, the location of the partically changed pixel in the key is random and the values are set to be zero. These results (very small maximum nonlinear cross-correlation values) indicate that the image can not be verified with an incorrect phase key, even though the incorrect and correct phase keys share many common values. This result guarantees the security of the proposed method against brute-force attacks.

## 6. Conclusions

In this paper, we have proposed a combination of multispectral photon-counting imaging (MPCI) and double random phase encoding (DRPE) for multispectral image authentication. Experimental results showed that the decrypted images from the proposed combination cannot be visually recognized with a limited number of photons and thus can provide an additional layer of security. Nevertheless, the primary image can be authenticated with the decrypted image using nonlinear cross-correlation metrics based on either a Bayer or a RGB color image. The procedure can also achieve better bandwidth reduction since the encrypted image is sparsely distributed. In addition, the proposed system can be robust even with partial encryption and under brute-force attacks. When the encrypted images are partially occluded or changed, correct authentication results can be achieved. However, if even a few values are altered in the correct phase-decryption key, image verification will fail. Experimental results also reveal that Malvar's demosaicing algorithm can obtain better authentication results than other methods based on multispectral RGB color images.
